# Clinical Yi-guan decoction for liver cirrhosis: A protocol for systematic review and meta analysis

**DOI:** 10.1097/MD.0000000000024530

**Published:** 2021-04-02

**Authors:** Xingyao Hu, Guangbin Shang, Jie Zhang, Zhong Chen, Liu Fu, Jun Li, Xiaonan Lu

**Affiliations:** a Research Center for Differention and Development of TCM Basic Theory; b The Affiliated Hospital of Jiangxi University of Traditional Chinese Medicine; c College of Traditional Chinese Medicine of Jiangxi University of Traditional Chinese Medicine, Nanchang, China.

**Keywords:** liver cirrhosis, protocol, systematic review and meta-analysis, Yi-guan decoction

## Abstract

**Background:**

At present, Liver Cirrhosis (LC) is common in most later liver and gallbladder diseases that its morbidity and mortality seriously affect human health. The limitation and effectiveness of western medicine on LC have become a huge clinical challenge. However, a large number of clinical studies have shown that Yi-guan decoction has become a complementary treatment for LC. Therefore, this systematic review will aim to explore the safety and feasibility of Yi-guan decoction in the treatment of LC.

**Methods:**

We will conduct a comprehensive literature search in Medline, PubMed, Cochrane Database of Systematic Reviews, Embase, Chinese Biomedical Literatures Database, China National Knowledge Infrastructure, Wang Fang Database, Chinese Scientific Journal Database from inception to December 2020 without any language restriction, In addition, relevant literature will be searched manually. The main subject terms searched: “Yi-guan decoction” “cirrhosis” “LC”. Data entry will be performed by 2 researchers separately. Primary outcomes will be concluded: Liver function indicators: Total bilirubin, Alanine transaminase, Aspartate aminotransferase, etc. Secondary outcome indicators: Total effective rate, Nutrition index, Survival analysis, Adverse events; All randomized controlled trials collected in this study will be evaluated and rated using the Cochrane risk-of-biasassessment tool. Meta-analysis will be performed using RevMan 5.4.0 software. The heterogeneity test will be conducted between the studies, *P* < .1 and I^2^ > 50% are the thresholds for the tests. Using solid effect model or random effect model will be based on its heterogeneity value.

**Results:**

This systematic review provides a theoretical basis for Yi-guan decoction to treat LC, we will report this result soon.

**Conclusion:**

This study will explore Yi-guan decoction can will be used as one of the non drug therapies to prevent or treat LC.

**Trial registration number:**

INPLASY2020120114.

## 1 Introduction

Liver cirrhosis (LC) is the terminal stage of various chronic liver diseases, the main pathological features are diffuse liver fibrosis and structurally abnormal nodules, abnormal blood vessels inside and outside the liver.^[1,2]^ It can be divided into 2 pathological states clinically: compensation period and decompensation period. Patients with compensated LC will not show obvious symptoms, but the decompensated phase can manifest as portal hypertension, abnormal liver function, and serious complications (ascites, hepatic encephalopathy, hepatorenal syndrome, etc).^[3,4]^

For clinical diagnosis of LC requires a comprehensive examination of the patient's symptoms, signs, tests and complications. However, early diagnosis of LC is difficult that the gold standard for diagnosis still depends on liver biopsy.^[5,6]^ Based on this, the specific pathogenesis of LC is constantly being explored, in order to find better diagnosis and treatment methods. The onset of LC is related to many factors. Extra-hepatic factors are mostly caused by smoking, drinking, obesity, and other chronic diseases (Chronic kidney disease, hyperlipidemia, etc). Intrahepatic factors are Hepatitis C Virus infection and alcoholic liver disease as the main pathological causes.^[7,8]^ The recognition of normal liver tissue to cirrhosis can be summarized as follows: From the degradation of hepatic extracellular matrix, local fibrous tissue accumulation, blood vessel damage, and the release of intracellular substances such as process, the most important central link is the activation of hepatic stellate cells. The pathological manifestation is that when the liver is damaged for the first time. The damaged area is wrapped by extracellular matrix or fibrocytes; the damage continues repeatedly, leading to the accumulation of fibrous material and gradually forming liver fibrosis or cirrhosis. At the same time, it triggers the release of inflammatory factors (Tumor Necrosis Factor-α, transforming growth factor-β, etc) to transform hepatic stellate cells into proliferative myofibroblast-like cells. Hepatic fibrosis progression to cirrhosis is due to the continuous remodeling of hepatic lobule structure and blood circulation.^[9–13]^

Currently, cirrhosis of the liver causes 1.2 million deaths worldwide each year, accounting for 3.5% of the world's deaths, ranking 10th in most developed countries. In more developed countries, cirrhosis is an increasing incidence and mortality rate, ranking 14th globally but fourth in Central Europe. The incidence in Asia ranges from 16.5 per 100,000 in East Asia to 23.6 per 100,000 in Southeast Asia. Therefore, more and more people believe that LC is not a single disease entity, but a disease that can be subdivided into different clinical prognostic stages. According to different stages, the mortality rate ranges from 1% to 57%.^[14–16]^

Although patients with decompensated LC undergo standardized treatment, their survival rate within 5 years is 14% to 35%. In this regard, the treatment of cirrhosis should be diagnosed and treated as early as possible. Pay attention to the treatment of the pathogeny, providing anti-inflammatory, anti-fiber, prevention, and treatment of complications when necessary. If the drug is not effective, surgical treatment such as artificial liver and intervention can be used. Of course, liver transplant surgery can also be applied.^[17–19]^ In the process of the treatment of cirrhosis, there is no cure for the fibrosis symptoms with western medicine. However, as complementary and alternative medicine. Traditional Chinese Medicine (TCM) is one of the safe and effective methods to treat LC. After the treatment with TCM, the survival rate of patients can be improved.^[20–23]^

Yi-guan decoction was created by Wei Yupu, a physician in the Qing Dynasty, from “Supplement to Classified Case Records of Celebrated Physicians”. It is composed of Dried Rehmannia Root, Radix Adenophorum, Ophiopogon, Angelica, wolfberry, and Fructus Toosendan. It is clinically used for patients with gan-shen-yin-xu deficiency diseases such as Hepatobiliary diseases, stomach diseases, diabetes, gynecological diseases and other diseases. Some doctors call it “the supreme cure for liver-yin”. Modern research has found that Yi-guan decoction has anti-fatigue, anti-hypoxia, anti-inflammation, enhance the phagocytic function of phagocytic cells, effectively improve liver histopathological changes, improve liver inflammation, increase the content of serum albumin, promote ascites regression, and other effects.^[24–26]^

At present, the reliability and feasibility of certain clinical methods are mainly evaluated by systematic evaluation.^[27]^ From the previous discussion, we can see that the number of patients with LC is increasing year by year, and the self-limiting nature of western medicine has gradually become prominent, but the theory of syndrome differentiation, holistic treatment and the effectiveness of TCM on patients with LC are gradually being explored. By consulting the literature, we can see that Yi-guan decoction has a significant effect on patients with LC that published literature has gradually increased. However, there is still a lack of systematic evaluation of the safety and effectiveness of Yi-guan decoction for LC. Therefore, it is better to serve the clinic, so the author has a deep excavation.

## 2 Methods and analysis

### 2.1 Research registration

This systematic review has been registered on the International Prospective Register of Systematic Reviews, Registration Number: INPLASY2020120114; Register URL: https://inplasy.com/inplasy-2020-12-0114/. At the same time, this article follows the corresponding guidelines (The protocol follows the Cochrane Handbook for Systematic Reviews and Meta-Analysis Protocol).^[28]^ Of course, this article does not need to implement ethical approval guidelines.

### 2.2 Eligibility criteria

#### 2.2.1 Research type

This systematic review will search the Chinese, English databases for clinical randomized controlled trials (RCT) of Yi-guan decoction in the treatment of LC. Of course, manual retrieval is required for unpublished literature. non-RCT must be excluded.

#### 2.2.2 Types of participants

The inclusion of this literature must be a RCT, and patients who meet the diagnostic criteria for LC^[2]^ will be included. This article does not limit the age, gender, and source of the patient that Exclude with other diseases (hypertension, diabetes) in patients with LC.

#### 2.2.3 Types of interventions

Patients with LC in the Test group must take Yi-guan Decoction as the main treatment (could be combined with other treatment methods or used alone), the control group can not use Yiguan Decoction;

#### 2.2.4 Type of comparators

In the control group, intervention means can include blank control, drug (TCM, western medicine) treatment, routine symptomatic treatment, etc.

#### 2.2.5 Types of outcome measures

##### 2.2.5.1 Primary outcomes

Liver function indicators: total bilirubin, Alanine transaminase, Aspartate aminotransferase, etc.

##### 2.2.5.2 Secondary outcome indicators

(1) total effective rate
(2) Nutrition index
(3) Survival analysis
(4) Adverse events;

### 2.3 Exclusion criteria

Document type is before and after comparison; In the control group, the Yi-guan decoction was used consistently; Non-randomized controlled studies such as repetitive literature, theoretical discussions, review literature, animal experimental studies.

### 2.4 Literature retrieval method

We will conduct a comprehensive literature search in Medline, PubMed, Cochrane Database of Systematic Reviews (Cochrane Library), Embase, Chinese Biomedical Literatures Database, China National Knowledge Infrastructure, Wan Fang Database, Chinese Scientific Journal Database (VIP) from inception to December 2020 without any language restriction. The main subject terms searched: “Yi-guan decoction” “cirrhosis” “LC”. The retrieval strategy of PubMed database is shown in Table 1. Other database retrieval methods will be adjusted in line with database differences.

**Table 1:** The search strategy for PubMed.

Order	Strategy
#1	Search “liver cirrhosis” [MeSH] or “liver cirrhosis, biliary” [MeSH] or “liver cirrhosis, alcoholic” [MeSH]
#2	Search “liver fibrosis” [Ti/Ab] or “cirrhosis” [Ti/Ab] or “hepatic cirrhosis”[Ti/Ab] or ”liver cirrhosis, biliary”[Ti/Ab] or “liver cirrhosis, alcoholic”[Ti/Ab] or “LC”[Ti/Ab]
#3	#1 Or #2
#4	Search “Yi Guan Jian Decoction “[Supplementary Concept]
#5	Search “Yi guan jian” [Ti/Ab] or “Yi guan jian decoction “ [Ti/Ab] or “Yi Guan decoction”[Ti/Ab]
#6	#4 and #5
#7	Search “Randomized controlled trial” [MeSH] or “controlled clinical trial” [MeSH]
#8	Search “Randomized controlled trial” [Ti/Ab] or “clinical trial” [Ti/Ab] or “randomized” [Ti/Ab]
#9	#7 or #8
#10	#3 and #6 and #9

### 2.5 Literature screening

#### 2.5.1 Screening process

We will deal with the conforming article in the following ways: First of all, search the databases which are the correct search method according to the search terms of the selected disease and treatment plan; Then import the retrieved article into noteexpress 3.0, in which preliminarily eliminate duplicate literature according to the software index; After reading the abstract and title of the remaining article, select the essay that meet the inclusion criteria; then download the remaining articles one by one, reading the full text, Selecting the final accepted article.

**Figure 1:**
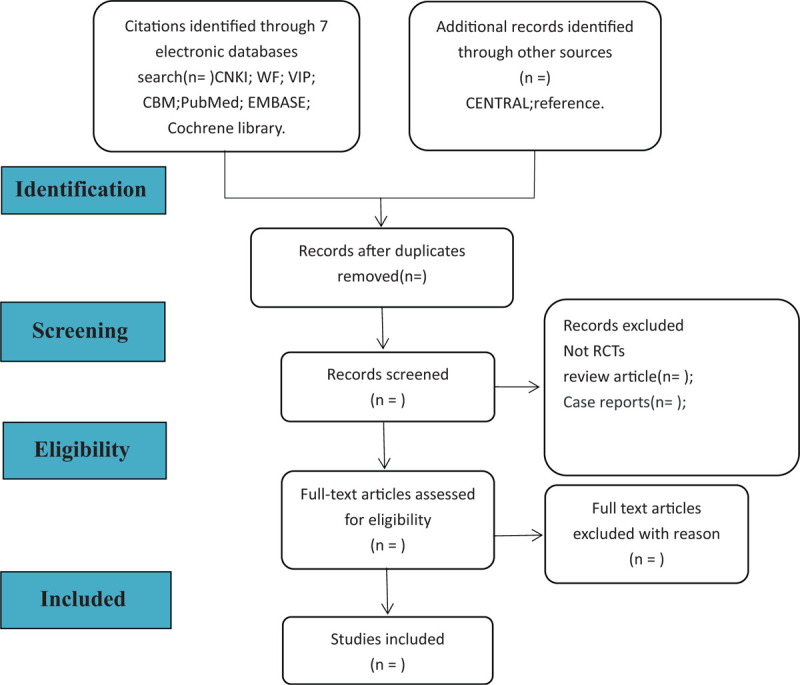
Flowchart of literature selection.

In the above process of screening articles, 2 researchers (Ge Liu, Zhong Chen) will strictly abide by the operating standards to screen essay. When 2 researchers disagree on an article, they can consult with a third party (Xingyao Hu). The process of including literature is shown in Figure 1.

#### 2.5.2 Information input

The included documents will be independently extracted by 2 researchers and entered into excel 2010. After the information extracted by the 2 researchers (Ge Liu, Zhong Chen) will be cross-checked. If there is a discrepancy, discussing with the third party (Xingyao Hu) to ensure the accuracy of the information. The included information includes: author, title, Publication Date, sample size, and Outcome indicators, etc. For some of the included literature information is missing, we will contact the author directly by phone or email.

#### 2.5.3 Methodological quality evaluation

The methodology for evaluating the quality and risk of bias will be completed with Cochrane Reviewer's Handbook 5.0.^[29]^ Its content includes the following 7 items: random method; allocation hiding; Blind implementation; result data integrity; outcome rater blinded; Selective reporting results; The above 6 indicators contain “yes”, “no” and “unclear”. Two evaluators need to make an evaluation in the corresponding options. If there is a dispute during the selection process, they can discuss with a third party (Xingyao Hu) deal with.

### 2.6 Data analysis integration

#### 2.6.1 Data synthesis

Data can be divided into counting data and measurement data. Counting data is expressed by odds ratio and 95% confidence interval; Weighted Mean Difference and 95% confidence interval is used to represent measurement data. Standardized Mean Difference is used when the units are not uniform.

#### 2.6.2 Description of heterogeneity

Heterogeneity is doing to test by I^2^. When *P* > .1 and I^2^ < 50%, the fixed-effect model will be adopted; When *P* < .1 and I^2^ > 50%, the random effects model will be used. When there will be greater heterogeneity that use sensitivity analysis. When there will be substantial heterogeneity, descriptive analysis can be used.

#### 2.6.3 Publication bias

If the number of included documents is large (>10), the software Review Manager 5.4 line inverted funnel graph can be used to analyze the bias situation that the shape of the graph can provide reference.

#### 2.6.4 Subgroup analysis

If there will be great heterogeneity in the analysis process, subgroup analysis of the included articles will be performed according to different control measures;

#### 2.6.5 Sensitivity analysis

The purpose of sensitivity analysis is going to evaluate the authenticity of meta-analysis. We will use software Stata 14.0 to perform sensitivity analysis.

## 3 Discussion

This systematic review will be based on the first summary of the treatment of livers cirrhosis by Yi-guan decoction in modern literature that its purpose is to find a strong evidence-based basis for the treatment of livers cirrhosis. For the discussion part of the next article, it will be discussed in the following directions:

1. The origin of Yi-guan decoction and the composition of the medicine;
2. Modern research on the effect of Yi-guan decoction on LC
3. Make a horizontal comparison with other intervention methods;
4. Discuss the results
5. Conclusion

The development of TCM has a history of thousands of years. The role of TCM in the treatment of LC has been gradually explored and gradually introduced as a complementary therapy for refractory diseases. Only by combining TCM and Western medicine can doctors make correct and reasonable clinical decisions.^[30–32]^ Studies have shown that Yi-guanjian has an effect on the activation of hepatic stellate cells and the regulation of various pathways that has been widely used in the treatment of patients with LC.^[33–35]^ Based on the current number of articles on the treatment of LC by Yi-guan decoction. Therefore, the purpose of this article is to provide the safety of Yiguan Decoction in the treatment of LC and to provide evidence-based medical advice. Of course, this study still has certain limitations: First, the low quality of the literature will affect the results of this systematic review; second, the severe language limitations of the literature will make the literature search incomplete; third, there may be missing data because the author of the article cannot be contacted. Therefore, to objectively evaluate the effectiveness and safety of Yi-guan decoction in the treatment of LC, a large number of clinical controlled trials and mechanism studies are still needed.

## Author contributions

**Conceptualization:** Xingyao Hu.

**Data curation:** Zhong Chen, Liu Fu.

**Formal analysis:** Guangbin Shang, Jie Zhang.

**Investigation:** Xingyao Hu, Jie Zhang.

**Methodology:** Guangbin Shang, Jun Li.

**Project administration:** Xiaonan Lu.

**Software:** Xingyao Hu, Jie Zhang.

**Supervision:** Xiaonan Lu.

**Validation:** Xiaonan Lu.

**Writing – original draft:** Xingyao Hu, Liu Fu.

**Writing – review & editing:** Guangbin Shang, Jie Zhang, Zhong Cheng, Liu Fu, Jun Li.
